# Decrease in Serum HDL-C Level Is Associated with Elevation of Blood Pressure: Correlation Analysis from the Korean National Health and Nutrition Examination Survey 2017

**DOI:** 10.3390/ijerph17031101

**Published:** 2020-02-09

**Authors:** Kyung-Hyun Cho, Hye-Jeong Park, Jae-Ryong Kim

**Affiliations:** 1 Korea Research Institute of Lipoproteins, Medical Innovation Complex, Daegu 41061, Korea; hjgod6867@naver.com; 2 LipoLab, Yeungnam University, Gyeongsan 712-749, Korea; 3 Department of Biochemistry and Molecular Biology, Smart-Aging Convergence Research Center, College of Medicine, Yeungnam University, Daegu 705-717, Korea; kimjr000@gmail.com

**Keywords:** high-density lipoproteins, cholesterol, blood pressure, dyslipidemia, hypertension

## Abstract

A low-serum, high-density lipoproteins–cholesterol (HDL-C) level and high blood pressure (BP) are independent risk factors for cardiovascular disease and dementia. In the present study, in order to find putative correlation between low HDL-C and hypertension, 4552 subjects (20–80 years old) were selected from the Korean National Health And Nutrition Examination Survey 2017 (KNHANES VII-2, *n* = 2017 men, *n* = 2535 women). They were classified into four levels of blood pressure, ranging from BP1 (normal, below 120/80 mmHg for systolic BP (SBP)/diastolic BP (DBP), BP2 (prehypertension, 120/80 to 139/89 mmHg), BP3 (hypertension stage 1, 140/90–159/99 mmHg), and BP4 (hypertension stage 2, higher than 160/100 mmHg). Generally, in the total population, a higher SBP level and age were associated with a lower HDL-C in both genders. However, DBP was not associated with age in men. In the total population, Pearson’s correlation analysis revealed that SBP (r = −0.188, *p* < 0.001) and DBP (r = −0.198, *p* < 0.001) showed negative correlations with percentage of HDL-C in total cholesterol (TC), HDL-C/TC (%). In both genders, HDL-C gradually decreased with age and HDL-C/TC (%) was more accurate in expressing a correlation with BP. Women showed a more distinct decrease in HDL-C with an elevation of BP and age than men. Both elevation of DBP and SBP were associated with a decrease in HDL-C, around 2.3–2.4 mg/dL, between normal range and hypertension 2 stage. Additionally, DBP was significantly associated with HDL-C/TC (%) (men: r = −0.136, *p* < 0.001; women: r = −0.152, *p* < 0.001), while HDL-C did not show a significant association with a change in DBP. In conclusion, SBP was positively correlated with age, but DBP did not change significantly with age. The correlation of BP and HDL-C depending on age showed that SBP gradually increased and HDL-C decreased with an increase in age. The percentage of HDL-C in TC was more significantly associated with a change in SBP and DBP in both genders.

## 1. Introduction

Many epidemiological studies revealed that there is a positive correlation between serum cholesterol and blood pressure (BP); hypertensive patients frequently showed higher cholesterol levels than normotensive subjects [1], although the putative mechanism of the link is still unknown. It has been well known that hypertension is an independent risk factor of cardiovascular disease [2], development of cognitive decline [3], and Alzheimer’s disease [4]. Dyslipidemia measures including high total cholesterol (TC) and low high-density lipoproteins-cholesterol (HDL-C) are also independent risk factors of cardiovascular disease and cerebrovascular disease [5]. However, there has been no correlation analysis between change in blood pressure and HDL-C in general population.

Hypertension in midlife is also a strong and independent risk factor of Alzheimer’s disease and vascular dementia in late-life [6,7], because individuals who develop hypertension earlier in life are likely to be exposed to the deleterious neurological effects of hypertension for many decades. The higher midlife BP was associated with incidence of dementia in late-life [6]. Interestingly, subjects with midlife hypertension and late-life hypotension had a 4.26-fold higher risk of dementia. Blood pressure control is very important to prevent not only cardiovascular disease but also incidence of cognitive impairment and dementia [8]. The positive association of TC and hypertension and inverse associations of HDL-C and future risk of hypertension are well established [9].

As well as high SBP in midlife, low DBP in late life is also dangerous because it is associated with smaller brain volumes and poor cognitive outcomes in older adults [10]. Many epidemiological studies revealed that blood pressure is prone to gradually elevating with increasing age [11], therefore, hypertension is more prevalent in the elderly population than the young population. However, global awareness of hypertension is less known: one-third of the total population in 90 countries did not know their own BP level, even though hypertension occurs in one third of the world’s adults and two-thirds of adults over age 65 [12].

Low HDL-C and high blood pressure are biomarkers for metabolic syndrome, as well as insulin resistance, abdominal obesity, and hypertriglyceridemia. Numerous reports and meta-analysis have provided evidence for increased risk of dementia in patients with metabolic syndrome. Recently, we reported that Korean adults showed a gradual decrease in HDL-C with increasing age from their 20s to 80s, while the incidence of dementia was sharply elevated after 60 years [13]. The women’s group showed a particularly sharp decrease in HDL-C with age, especially in those older than 50 years, then incidence of dementia was dramatically elevated after 60 years. The decrease in HDL-C after middle age was strongly associated with the considerable increase in dementia in late-life. It has been well known that controlling BP in the normal range is an effective strategy for the prevention of primary and secondary dementia [14]. However, there has been no study to elucidate the correlation and regression between blood pressure and the HDL-C level in the adult population.

In the current study, we investigated the cross-sectional relationship between BP and serum HDL-C in the free-living Korean population because there has been no report of the correlation between HDL-C level and SBP or DBP. This study analyzed the correlations of TC, HDL-C and HDL-C/TC (%) level with SBP and DBP from 2017 KNHANES. The aim of this study is to provide statistical evidence for the logical relationships among HDL-C, HDL-C/TC (%), SBP, and DBP depending on age and gender.

## 2. Methods

The current research was based on the Seventh Korean National Health and Nutrition Examination Survey in 2017 (KNHANES VII-2, approval number 117002), performed by the Korea Centers for Disease Control and Prevention (KCDC). The KNHANES is a nationwide, population-based, cross-sectional survey conducted by the Division of Chronic Disease Surveillance of the KCDC to examine the health and nutritional status of the population [15]. Trained interviewers collected all data using structured questionnaires and obtained data regarding the education attainment, socioeconomic status, health, lifestyle and laboratory information, as well as the male and female reproductive history.

### 2.1. Study Population

**Figure 1:**
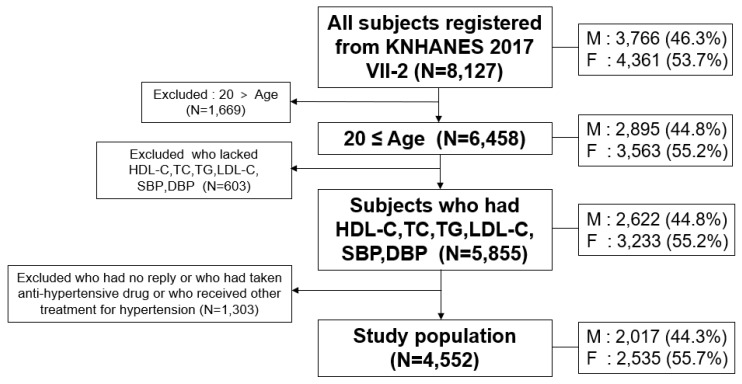
Inclusion criteria and subject number in analysis from KNHANES, Korean national health and nutrition examination survey; HDL-C, high-density lipoprotein cholesterol; TC, total cholesterol; TG, triglyceride; LDL-C, low-density lipoprotein-cholesterol; SBP, systolic blood pressure; DBP, diastolic blood pressure; M: men; F: female.

As a health examination survey, the initial number of subjects in the KNHANES VII-2 (*n* = 8127) were examined by laboratory measurements for serum cholesterol and answered questionnaires on health and nutrition. We excluded participants aged less than 20 years (*n* = 1669). Subjects (*n* = 603) were also excluded if they lacked information on HDL-C, TC, TG, LDL-C, systolic SBP (SBP), and diastolic BP (DBP). Then, we excluded patients (*n* = 1303) who had taken/were currently on anti-hypertensive drugs or who received other treatments for hypertension, and subjects who did not reply to the question about treatments for hypertension. The final study population was 4552 individuals (2017 men, 2535 women) based on HDL-C and BP, as shown in Figure 1, who were sampled randomly throughout South Korea. Adult subjects, 20–80 years old, who reported their BP and blood biochemistry data, including total cholesterol (TC) and HDL-C, were selected. A low HDL-C level in men and women was defined as <40 and <50 mg/dL, respectively, according to the guidelines of the National Cholesterol Education Program-Adult Treatment Panel III [16].

They were classified into four levels of blood pressure, ranging from BP 1 (normal, *n* = 2905, normal, below 120/80 mm Hg for SBP/DBP), BP 2 (prehypertension, *n* = 1280, 120/80 to 139/89 mmHg), BP 3 (hypertension stage 1, *n* = 313, 140/90-159/99 mmHg), and BP 4 (hypertension stage 2, *n* = 54, higher than 160/100 mmHg). Self-reported questionnaires were given to the subjects to determine their education period, household income, residential area, marital status, education level, occupation, economic status, smoking, alcohol intake, and physical activity. The serum TC, HDL-C, and LDL-C were measured directly by a homogeneous enzymatic method using Pureauto SCHO-N, Cholest N HDL, and Cholestest LDL agent (Sekisui, Japan), respectively, with a Hitachi Automatic Analyzer 7600-210 (Hitachi, Japan).

### 2.2. Statistical Analysis

All values are expressed as the mean ± SD (standard deviation) for the continuous variables. All analyses were normalized by a homogeneity test of variances through Levene’s statistics. The continuous levels of lipid parameters, such as HDL-C, TC, TG and SBP, DBP, and age, were compared using independent samples *t*-test depending on gender, as seen in Table 1. For subcategory analysis, each ranging of BP and age group were shown as a percentage and compared using independent samples *t*-test depending on gender, as seen in Tables 2 and 3.

The differences in the serum concentrations of HDL-C, HDL-C/TC (%), and age among the four categories of BP were determined using ANOVA. The differences in the serum concentrations of HDL-C, HDL-C/TC (%), SBP, DBP by age were determined using ANOVA. The Bonferroni and Games-Howell post-hoc test was used to determine the significance of the differences in the continuous variables to identify the differences between each category (BP and age). A multiple group test procedure was performed to determine the pattern, with either an increase or decrease in HDL-C, HDL-C/TC (%), age, SBP and DBP, according to Jonckheere–Terpstra (J–T test), as described previously [17,18]. All graphs performed Pearson’s correlation analysis to find a positive or negative association. Linear regression analysis was carried out to evaluate the independent factor of HDL-C to influence change in SBP or DBP (dependent factor) in total population, adjusted with age variables to exclude influence of BP.

All tests were two-tailed and the statistical significance was defined at *p* < 0.05. Statistical analyses were carried out using the SPSS statistical package version 25.0 (SPSS Inc., Chicago, IL, USA) incorporating sampling weights and adjusting for the complex survey design of the KNHANES 2017.

### 2.3. Ethics Statement

Korea National Health and Nutrition Examination Survey (KNHANES) is an annual review and has been approved by KCDC Research Ethics Review Committee since 2007 (approval no. 2013–12EXP03–5C). The committee operates under the KCDC Research Ethics Review Committee’ s standard guidelines based on domestic and international regulations and guidelines, such as the Declaration of Helsinki and the Bioethics and Safety Act. Informed consent was obtained from all participants when the surveys were conducted.

## 3. Results

### 3.1. General Profiles of BP and Lipid

On average, as shown in Table 1, the total population (*n* = 4552) showed a normotensive (SBP, 116.1 ± 15.8 mmHg; DBP, 75.5 ± 10.0 mmHg) and normolipidemic profile with midlife age (47.6 ± 15.4 year-old). There was no difference in age between men and women (*p* = 0.988). Men had 4.9% and 6.5% higher SBP and DBP, respectively, than women (*p* < 0.001). Although there was no difference in TC levels between men and women (*p* = 0.103), men showed 14.4%- and 3.9%-point lower HDL-C and HDL-C/TC (%) level, respectively, than women. These results suggest that higher BP and lower HDL-C is coincident in men, with no difference in TC level and age. The total population showed a normal range of serum TC, TG, and LDL-C. Men showed 1.47-fold higher TG levels and a 1.72-fold higher TG/HDL-C ratio than women. As shown in Table 1, men had lower LDL-C levels (*p* = 0.00006) but a higher LDL-C/HDL-C ratio (*p* < 0.001) compared to women.

### 3.2. Distribution of BP, Age, and HDL-C in Total Population

Generally, as shown in Table 2, 63.8% of subjects were in normotension and 28.1% of subjects were in prehypertension, with 8.1% of patients with hypertension in the total population. In both SBP and DBP, a smaller percentage of men were normotensive subjects, with 56.2% and 57.4%, respectively, than women with 69.9% and 76.5%, respectively. Interestingly, percentages of prehypertension subjects in SBP or DBP were more increased in men, with 34.6% and 31.0%, respectively, than women, with 22.9% and 18.2%, respectively (Table 2).

**Figure 2:**
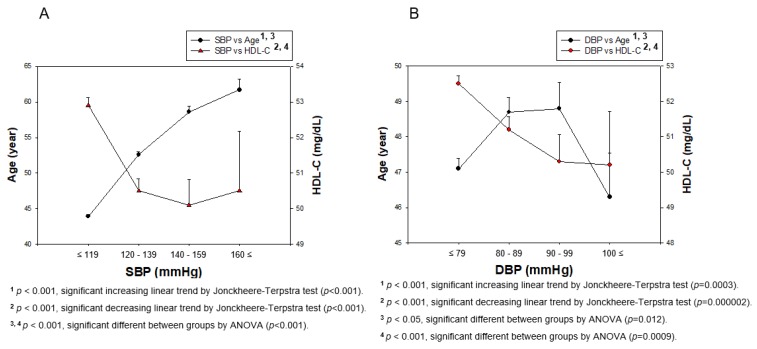
Correlation of age and HDL-C depends on SBP (**A**) and DBP (**B**) in total population. Negative association was found between age and HDL-C depends on SBP or DBP by J-T test. Pearson’s correlation analysis was carried out between age, HDL-C, and SBP (**A**) and DBP (**B**). Panel A showed positive correlation (r = 0.360, *p* < 0.001) between SBP and age; panel B showed positive correlation (r = 0.049, *p* = 0.0009) between DBP and age. In addition, panel A showed negative correlation (r = −0.116, *p* < 0.001) between SBP and HDL-C; panel B showed a negative correlation (r = −0.079, *p* < 0.001) between DBP and HDL-C.

DBP was not dependent on an increase in age. In both genders, HDL-C was decreased with an increase in SBP (*p* < 0.001) and DBP (*p* = 0.000002), while SBP (*p* < 0.001) was significantly elevated with increasing age (Figure 2). However, a higher DBP was not associated with increasing age, although HDL-C levels were negatively associated with DBP, as shown in Figure 2B. The difference between the SBP (*p* < 0.001) and DBP (*p* = 0.012) category regarding age was found to be significant using ANOVA. The difference between the SBP (*p* < 0.001) and DBP (*p* = 0.0009) categories from HDL-C was found to be significant using ANOVA. In total population, the elevation of SBP was associated with increasing age, although the association between HDL-C and SBP was different among men and women.

### 3.3. Distribution of BP and HDL-C Depends on Gender

**Figure 3:**
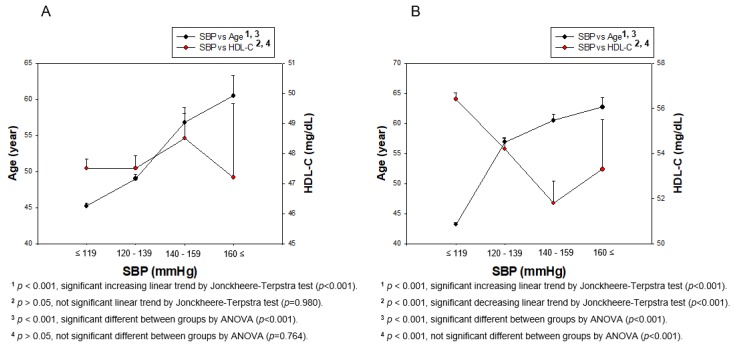
Correlation of age and HDL-C depends on SBP in men (**A**) and women (**B**). A negative association was found between age and HDL-C depending on SBP in women (**B**) by J–T test. Pearson’s correlation analysis was carried out between age, HDL-C, and SBP in men (**A**) and women (**B**). The men (**A**) showed r = 0.233 (*p* < 0.001) and women (**B**) showed r = 0.468 (*p* < 0.001) between SBP and age. Men (**A**) showed r=0.008 (*p* = 0.713) and women (**B**) showed r = −0.111 (*p* < 0.001) between SBP and HDL-C.

Men had almost the same distribution of HDL-C levels around 47.5–48.5 mg/dL (*p* = 0.980) through the entire range of SBP, while women had a sharply decreased HDL-C pattern around at 56.4 ± 12.0 and 51.8 ± 12.3 mg/dL (*p* < 0.001) for normotension and hypertension stage 1, respectively (Figure 3). Interestingly, the hypertension stage 2 category in women showed an even increase in HDL-C levels, although it showed no significance (*p* = 0.931). Men and women showed different ages with increasing SBP (men, *p* < 0.001; women, *p* < 0.001), as shown in Figure 3A,B. Moreover, women showed significant difference in HDL-C (*p* < 0.001), as shown in Figure 3B.

**Figure 4:**
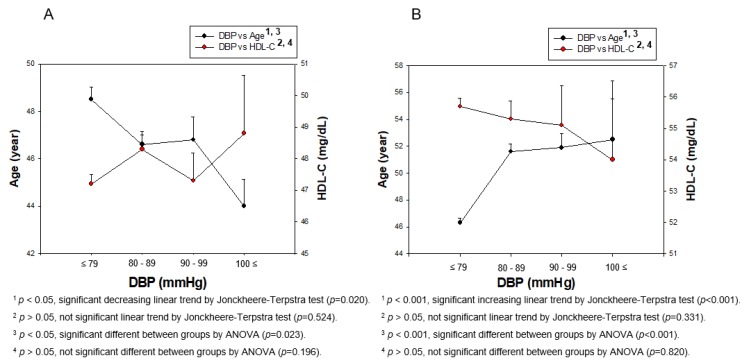
Pearson’s correlation analysis was carried out among age, HDL-C, and DBP in men (**A**) and women (**B**). The men (**A**) showed r = −0.079 (*p* = 0.0004) and women (**B**) showed r = 0.167 (*p* < 0.001) between DBP and age. However, men (**A**) showed r = 0.026 (*p* = 0.248) and women (**B**) showed r = –0.024 (*p* = 0.221) between DBP and HDL-C.

As shown in Figure 4A, men showed a decrease in age with elevation of the DBP category, (Table 2 and Figure 4A). However, women showed increase in age from 46.3±15.6 to 52.5 ± 14.0 years, depending on the elevation of the DBP category (*p* < 0.001), while HDL-C levels gradually decreased from 55.7 ± 12.0 to 54.0 ± 11.5 mg/dL, although there was no significance (*p* = 0.331, Figure 4B). Although men and women showed a significant increase in age the during elevation of the DBP (men, *p* < 0.023; women, *p* < 0.001), there was no significant difference in HDL-C levels during elevation of DBP (men, *p* = 0.196; women, *p* = 0.820), as shown in Figure 4A,B.

**Figure 5:**
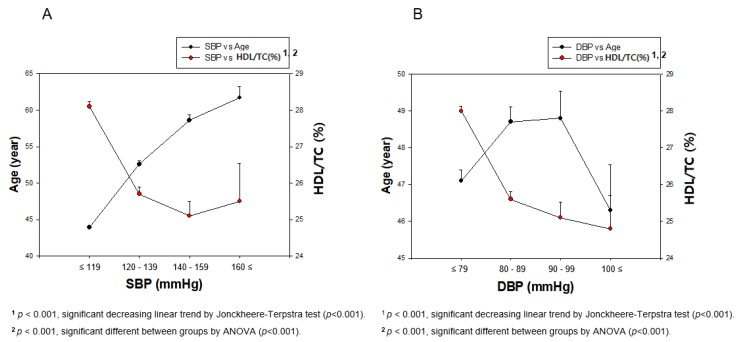
Correlation of age and HDL-C/TC (%) depends on SBP (**A**) and DBP (**B**) in total population. Negative association was found between age and HDL-C, depending on SBP or DBP, by J–T test. Pearson’s correlation analysis was carried out between age, HDL-C/TC (%), and SBP (**A**) and DBP (**B**). Panel A showed a negative correlation (r = −0.188, *p* < 0.001) between SBP and HDL-C/TC (%); panel B showed a negative correlation (r = −0.198, *p* < 0.001) between DBP and HDL-C/TC (%).

As shown in Figure 5, in the total population, HDL-C/TC (%) is more negatively associated with SBP (r = −0.188, *p* < 0.001) and DBP (r = −0.198, *p* < 0.001) than HDL-C (mg/dL), as indicated in Figure 2. Moreover, the elevation of DBP is more specifically correlated with a decrease in HDL-C/TC (%) than SBP, coincident with HDL-C (mg/dL).

**Figure 6:**
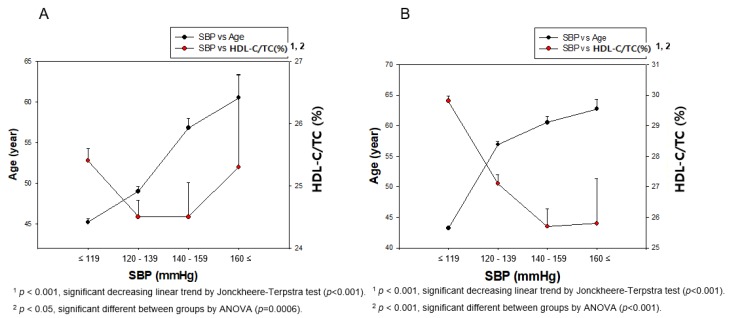
Correlation of age and HDL-C/TC (%) depends on SBP in men (**A**) and women (**B**). Negative association was found between age and HDL-C/TC (%) depends on SBP in women (**B**) by J–T test. Pearson’s correlation analysis was carried out between age, HDL-C/TC (%), and SBP in men (**A**) and women (**B**). Men (**A**) showed r = −0.065 (*p* = 0.003) and women (B) showed r = −0.208 (*p* < 0.001) between SBP and HDL-C/TC (%).

In the SBP, as shown in Figure 6, the HDL-C/TC (%) level of men was gradually decreased from normal range (25.4 ± 6.7%) to hypertension stage 1 (24.5 ± 6.9%) although HDL-C/TC (%) was slightly increased at hypertension stage 2 (25.3 ± 7.5%). However, women showed a sharp decrease in HDL-C/TC (%) from the normal range (29.8 ± 6.9%) to hypertension stage 1 (25.7 ± 7.1%, *p* < 0.001). Interestingly, hypertension stage 2 category in women showed almost the same HDL-C/TC (%) level (25.8 ± 7.9%) with hypertension stage 1. Generally, women showed a more distinct negative association (r = −0.065, *p* = 0.003) between HDL-C/TC (%) and SBP than that of men (r = −0.208, *p* < 0.001).

**Figure 7:**
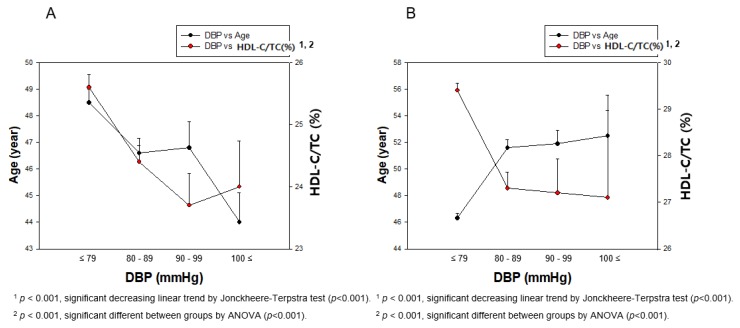
Pearson’s correlation analysis was carried out among age, HDL-C/TC (%), and DBP in men (**A**) and women (**B**). Men (**A**) showed r = −0.136 (*p* < 0.001) and women (**B**) showed r = −0.152 (*p* < 0.001) between DBP and HDL-C/TC (%).

In the DBP, as shown in Figure 7, the HDL-C/TC (%) level of men gradually decreased from normal range (25.6 ± 6.9%) to hypertension stage 1 (23.7 ± 6.8%) although HDL-C/TC (%) was slightly increased at hypertension stage 2 (24.0 ± 7.4%). However, women showed a sharp decrease in HDL-C/TC (%) from normal range (29.4 ± 6.9%) to prehypertension stage (27.3 ± 7.5%). After this, HDL-C/TC (%) was gradually decreased to (27.1 ± 8.6%) at hypertension stage 2 with the elevation of DBP. Interestingly, hypertension stage 2 in women showed almost the same HDL-C/TC (%) levels as hypertension stage 1. Generally, women showed a more distinct negative association (r = −0.152, *p* < 0.001) between HDL-C/TC (%) and DBP than that of men (r = −0.136, *p* < 0.001).

### 3.4. Lifetime Change in BP, HDL-C, and HDL-C/TC (%)

**Figure 8:**
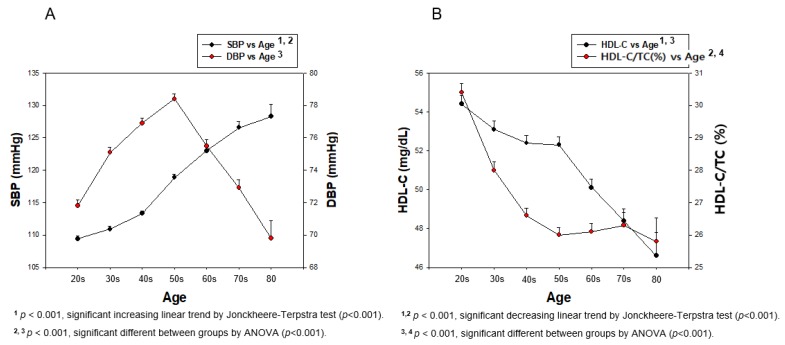
Change in BP and HDL-C depends on age in total population during entire life. Direct association was found between HDL-C and HDL-C/TC (%) depending on age by J–T test. Pearson’s correlation analysis was carried out among SBP, DBP, and age (**A**). Pearson’s correlation analysis was carried out among HDL-C, HDL-C/TC (%), and age (**B**). Panel A showed a negative correlation (r = −0.136, *p* < 0.001) between HDL-C and age; panel B showed a stronger negative correlation (r = −0.175, *p* < 0.001) between HDL-C/TC (%) and age.

Lifetime change in BP revealed that the total population showed a gradual increase in SBP, around 1.17-fold, from their 20s (109.4 ± 11.3 mmHg) to their 80s (128.3 ± 17.6 mmHg), as shown in Table 3 (*p* < 0.001). DBP was elevated until 50s in both genders, after that, it was gradually decreased until their 80s, as shown in Table 2 and Figure 8. However, DBP from the total population showed a 9% increase until age reached the 50s from the 20s, and then gradually decreased to less than the 20s’ DBP (Table 3 and Figure 8A). The serum HDL-C level of the total population showed a gradual decrease tendency from the 20s (54.4 ± 12.0 mg/dL) to the 80s (46.6 ± 11.3 mg/dL, *p* < 0.001). In the same manner, the HDL-C/TC (%) of the total population was also decreased from 30.4 ± 7.2% at the 20s to 25.8 ± 6.8 at the 80s (*p* < 0.001), as shown in Table 3 and Figure 8B. The difference in age category from SBP (*p* < 0.001) and DBP (*p* < 0.001) in the total population was found to be significant using ANOVA. The difference between age category from HDL-C (*p* < 0.001) and HDL-C/TC (%) (*p* < 0.001) in the total population was found to be significant using ANOVA.

**Figure 9:**
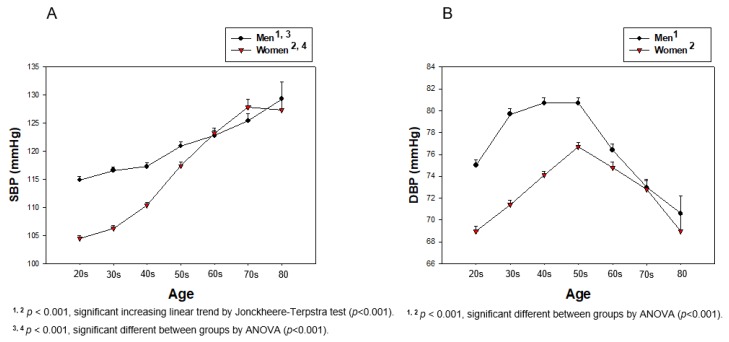
Change in SBP (**A**) and DBP (**B**) depends on age of men and women during entire life.

Men in their 20s showed 10.4 and 6.0 mmHg higher SBP and DBP, respectively, than women, and the gap was kept until 60 years. However, both groups showed the same SBP and DBP after 60 and 70, respectively. In men, SBP was 12.5 % increased (*p* < 0.001) and DBP was 5.9% decreased from their 20s to their 80s. Especially, the DBP was 7.6% increased from 20s to 50s, then gradually decreased by up to 12.5% from their 50s to 80s. Women showed a 21.8% increase in SBP from their 20s to 80s (*p* < 0.001). However, DBP was 11.2% increased from their 20s to 50s (*p* < 0.001), then 10.0% decreased until their 80s, as shown in Table 3 and Figure 9. The difference between age category from SBP (*p* < 0.001) and DBP (*p* < 0.001) in each gender was found to be significant using ANOVA.

**Figure 10:**
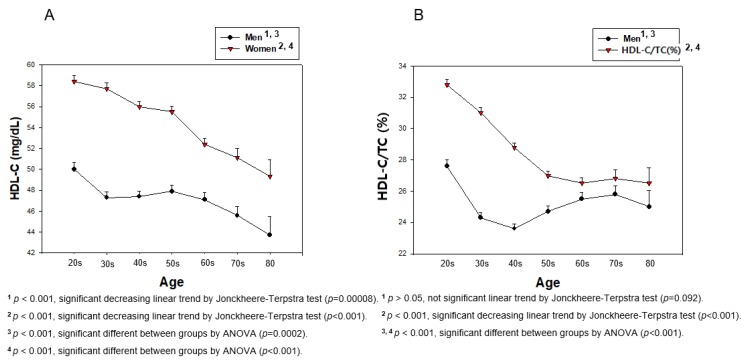
Change in HDL-C (**A**) and HDL-C/TC (%) (**B**) depends on age in men and women during entire life. A direct association was found of SBP depending on age in panel A by J–T test. Pearson’s correlation analysis was carried out between age and HDL-C (**A**) and HDL-C/TC (%) (**B**). Panel A showed r = −0.090 (*p* = 0.00005) in men and r = −0.184 (*p* < 0.001) in women between HDL-C and age. Panel B showed r = −0.037 (*p* = 0.100) in men and r = −0.297 (*p* < 0.001) in women between HDL-C/TC (%) and age.

In men, around 6.3 mg/dL of HDL-C levels was decreased from their 20s (50.0 ± 11.2 mg/dL) to their 80s (43.7 ± 11.5 mg/dL, *p* = 0.000008), while HDL-C/TC (%) was also 2.6%-point decreased although this was not significant (*p* = 0.092) from the 20s (27.6 ± 7.2 %) to 80s (25.0 ± 6.7 %). Women showed a more remarkable decrease in HDL-C level, from around 9.1 mg/dL (*p* < 0.001), and % HDL-C levels from around 6.3%-point (*p* < 0.001), from their 20s to 80s than men. The difference between age category from HDL-C (*p* < 0.001) and HDL-C/TC (%) (*p* < 0.001) in each gender was found to be significant using ANOVA. These results suggest that HDL-C/TC (%) level is more accurate, especially in women, to express change in HDL-C levels, which may explain the correlation, as shown in Table 3 and Figure 10.

### 3.5. Correlation of BP and HDL-C

In total population, HDL-C level showed a weak negative correlation with SBP (r = −0.116, *p* < 0.001) and DBP (r = −0.079, *p* < 0.001), as shown in Figure 2. Between HDL-C and SBP, as shown in Figure 3, men did not show a correlation (r = 0.008, *p* = 0.713), while women showed a weak correlation with significance (r = −0.111, *p* < 0.001). HDL-C level and DBP did not show any correlation among both genders (Figure 4).

Linear regression analysis revealed that a 1 mg/dL increase in HDL-C caused a decrease in SBP and DBP of 0.148 mmHg (*p* < 0.001) and 0.065 mmHg (*p* < 0.001), respectively (Table 4). HDL-C influenced negative associations with SBP (t = −7.880, *p* < 0.001) when adjusted with age variable. HDL-C also had a negative association with DBP (t = −5.376, *p* < 0.001). These results suggested that HDL-C had a stronger negative association with SBP than DBP.

### 3.6. Correlation of BP and HDL-C/TC (%)

Pearson’s correlation analysis revealed that SBP (r = −0.188, *p* < 0.001) and DBP (r = −0.198, *p* < 0.001) showed stronger negative correlations between BP and HDL-C/TC (%), as shown in Figure 5. Between HDL-C/TC (%) and SBP, as shown in Figure 6, men showed a negative correlation (r = −0.065, *p* = 0.003), while women showed a stronger negative correlation (r = −0.208, *p* < 0.001). Moreover, the correlation between HDL-C/TC (%) and DBP showed a stronger negative correlation (men, r = −0.136, *p* < 0.001; women, r = −0.152, *p* < 0.001) than that of HDL-C/TC (%) and SBP, as shown in Figure 7.

Pearson’s correlation analysis revealed that SBP and DBP showed negative correlations between BP and HDL-C/TC (%) in total cholesterol (TC), while SBP and DBP showed weaker negative correlations between BP and HDL-C.

### 3.7. Correlation Analysis Among age, HDL-C and HDL-C/TC (%)

In the total population, HDL-C level was negatively correlated with age (r = −0.136, *p* < 0.001) and HDL-C/TC (%) was more negatively correlated with age (r = −0.175, *p* < 0.001, Figure 8). In men, HDL-C level showed a weak negative correlation with age (r = −0.090, *p* = 0.00005) and HDL-C/TC (%) was less negatively correlated with age, although there was no significance (r = −0.037, *p* = 0.100, Figure 10). In women, HDL-C level had a strong negative correlation with age (r = −0.184, *p* < 0.001) and HDL-C/TC (%) had a stronger negative correlation with age (r = −0.297, *p* < 0.001, Figure 10).

### 3.8. Correlation of BP and TC

**Figure 11:**
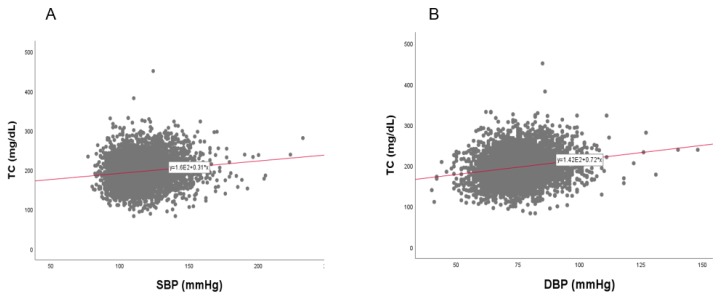
Pearson’s correlation analysis between total cholesterol (TC) and systolic blood pressure (SBP, panel **A**) and diastolic blood pressure (DBP, panel **B**) in total population (*n* = 4552). Panel **A** showed a positive correlation (r = 0.134, *p* < 0.001) between SBP and TC. Panel **B** showed a positive correlation (r = 0.196, *p* < 0.001) between DBP and TC.

**Figure 12:**
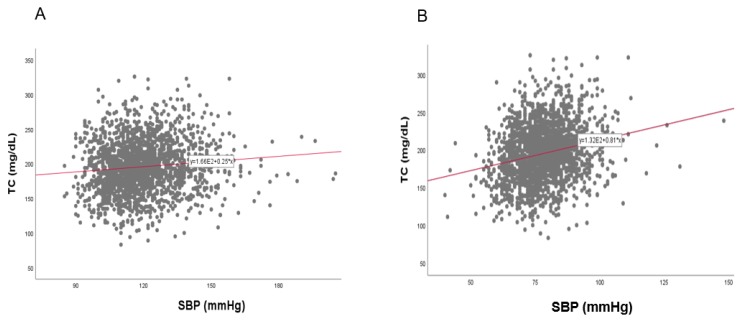
Pearson’s correlation analysis between total cholesterol (TC) and systolic blood pressure (SBP) in men (**A**) and women (**B**). The men (**A**) showed r = 0.101 (*p* = 0.000006) and women (**B**) showed r = 0.170 (*p* < 0.001) between SBP and TC.

**Figure 13:**
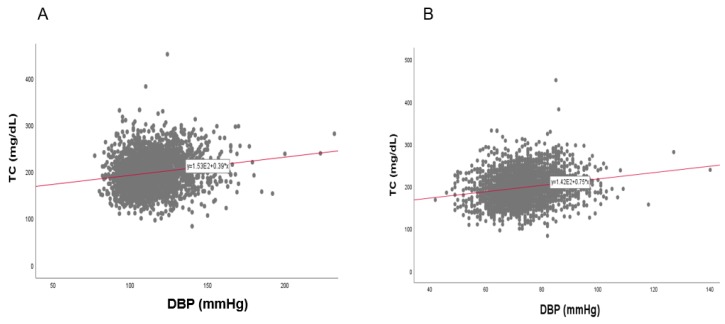
Pearson’s correlation analysis between total cholesterol (TC) and diastolic blood pressure (DBP) in men (**A**) and women (**B**). The men (**A**) showed r = 0.226 (*p* < 0.001) and women (**B**) showed r = 0.192 (*p* < 0.001) between DBP and TC.

In the total population, a strong positive correlation was found between SBP (r = 0.134, *p* < 0.001) and DBP (r = 0.196, *p* < 0.001) with TC, as shown in Figure 11. Between TC and SBP, as shown in Figure 12 and Figure 13, men showed a positive correlation (r = 0.101, *p* = 0.000006) and women also showed a positive correlation (r = 0.170, *p* < 0.001). Between TC and DBP, men showed a strong positive correlation (r = 0.226, *p* < 0.001) and women showed a positive correlation (r = 0.192, *p* < 0.001).

### 3.9. Correlation of BP and TG

**Figure 14:**
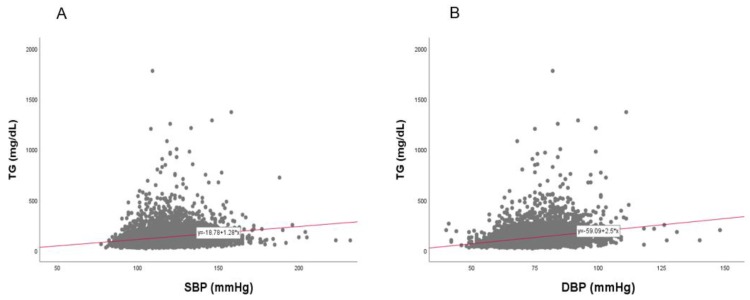
Pearson’s correlation analysis between triglyceride (TG) and systolic blood pressure (SBP, panel **A**) and diastolic blood pressure (DBP, panel **B**) in total population (*n* = 4552). Panel **A** showed a positive correlation (r = 0.192, *p* < 0.001) between SBP and TG; Panel **B** showed a positive correlation (r = 0.240, *p* < 0.001) between DBP and TG.

**Figure 15:**
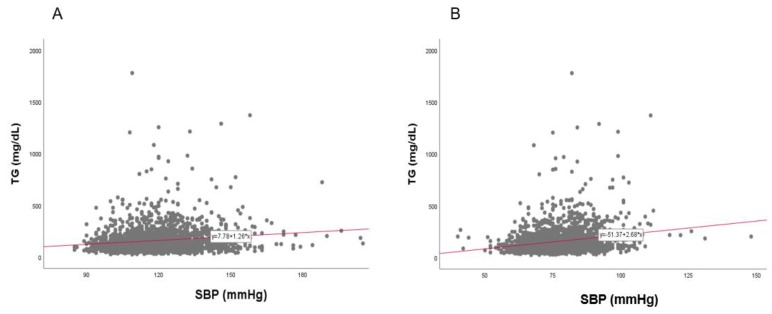
Pearson’s correlation analysis between triglyceride (TG) and systolic blood pressure (SBP) in men (**A**) and women (**B**). The men (**A**) showed r = 0.144 (*p* < 0.001) and women (**B**) showed r = 0.211 (*p* < 0.001) between SBP and TG.

**Figure 16:**
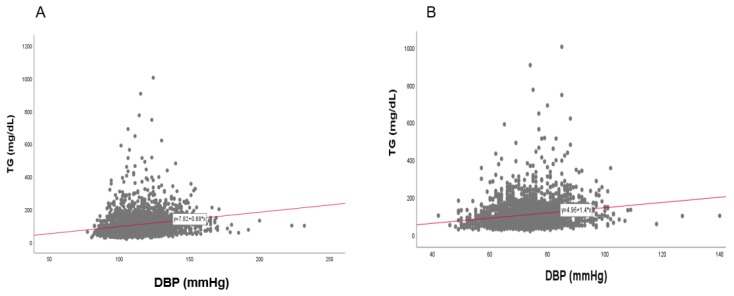
Pearson’s correlation analysis between triglyceride (TG) and diastolic blood pressure (DBP) in men (**A**) and women (**B**). The men (**A**) showed r = 0.193 (*p* < 0.001) and women (**B**) showed r = 0.180 (*p* < 0.001) between DBP and TG.

In the total population, a strong positive correlation was found between SBP (r = 0.192, *p* < 0.001) and DBP (r = 0.240, *p* < 0.001) with TG, as shown in Figure 14. Between TG and SBP, as shown in Figure 15 and Figure 16, men showed a strong positive correlation (r = 0.144, *p* < 0.001) and women showed a positive correlation (r = 0.211, *p* < 0.001). Between TG and DBP, men showed a strong positive correlation (r = 0.193, *p* < 0.001) and women showed a positive correlation (r = 0.180, *p* < 0.001).

**Figure 17:**
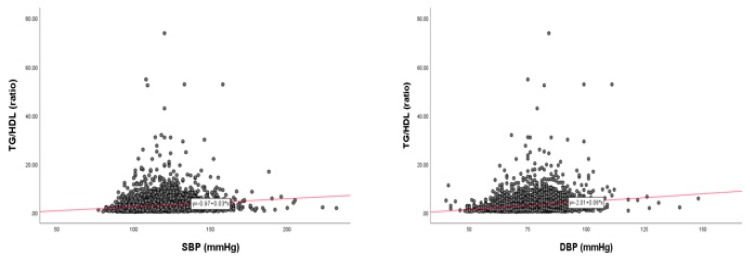
Pearson’s correlation analysis between TG/HDL ratio and systolic blood pressure (SBP, panel **A**) and diastolic blood pressure (DBP, panel **B**) in total population (*n* = 4552). Panel **A** showed a positive correlation (r = 0.156, *p* < 0.001) between SBP and TG/HDL ratio; Panel **B** showed a positive correlation (r = 0.194, *p* < 0.001) between DBP and TG/ HDL ratio.

In total population, correlation analysis between the TG/HDL-C ratio and SBP/DBP revealed a positive correlation between TG/HDL-C and SBP (r = 0.156, *p* < 0.001) and TG/HDL-C and DBP (r = 0.194, *p* < 0.001), as shown in Figure 17.

## 4. Discussion

This study focused on finding correlations of change among BP, HDL-C, and age. The major findings of the current study are: (1) total population showed a gradual elevation of SBP from ~20 to ~80 years of age; (2) the elevation of SBP is well correlated with a decrease in HDL-C during the entire life; (3) women showed a sharper decrease in HDL-C with the increase in SBP and age than men.

Interestingly, an increase in SBP was dependent on increase of age, but the increase in DBP was not dependent on age (Figure 9). The distribution of BP and HDL-C by increasing age showed that SBP was gradually increased and HDL-C was decreased in an age-dependent manner. A percentage of HDL-C in TC was more significantly associated with change of SBP and DBP in both genders. However, DBP was elevated only from the 20s to 50s, then decreased until the 80s, suggesting that SBP and DBP showed a different tendency of BP change with age. Although men had a higher SBP and DBP in their 20s, both men and women showed similar SBPs and DBPs in their 80s. The total population showed a negative correlation of SBP and DBP with HDL-C. While men did not show a correlation between HDL-C and SBP, women showed a significant negative correlation (Figure 3). In both men and women, there was no significant decrease in HDL-C with increasing DBP (Figure 4).

HDL-C/TC (%) was decreased with an increase of BP and age in both men and women, although women showed a sharper decrease from their 20s to 80s. HDL-C/TC (%) (r = −0.188, *p* < 0.001) was more significantly associated with an increase in SBP than HDL-C (r = –0.116, *p* < 0.001). HDL-C/TC (%) showed stronger negative correlations than HDL-C (mg/dL) between either SBP or DBP. In both genders, HDL-C gradually decreased with age and HDL-C/TC (%) was more accurate in expressing a correlation with BP. In the total population, HDL-C and HDL-C/TC (%) was gradually decreased from ~20 to ~80 years of age, although HDL-C/TC (%) had a sharper decrease than HDL-C. Between men and women, the difference in HDL-C at age ~20 was maintained until ~80 years of age; women showed higher HDL-C levels (Figure 10A). However, the difference in HDL-C/TC (%) in the 20–30 age range almost disappeared by 60–70 years, suggesting that HDL-C/TC (%) is more accurate and specific to express a correlation between SBP and DBP. Recently, Sydney-based Australian studies showed that exceptionally long-lived (ELL) individuals (95–106 years old) had significantly higher HDL-C/TC (%) (31.0%), while control individuals showed 25.9%. However, the ELL group and control group showed a normal range of HDL-C of 56.8 ± 17.4 mg/dL and 52.6±14.3 mg/dL, respectively, without a difference between the groups (9). Furthermore, the Prevention of Renal and Vascular End-stage Disease (PREVEND) prospective cohort study [19] revealed that HDL-C/TC (%) is significantly higher in the control group (around 26.3%) than the hypertension group (around 22.8%, *p* < 0.0001), although both groups showed s normal range of HDL-C at around 51–55 mg/dL. In the current study, women showed 4.7% and 6.2% less SBP and DBP than men, but 15.6% higher HDL-C/TC (%) (28.9 ± 7.1%) than men (25.0 ± 6.9%). Taken together, these results justify the use of HDL-C/TC (%) as a more suitable measure than HDL-C (mg/dL). Similarly, premenopausal systemic lupus erythematosus patients showed significantly lowered HDL-C/TC (%) (27 ± 3%) compared with healthy controls (32 ± 1%), with higher TC and lower HDL-C [20]. In the same context, recent data on Chinese patients with Alzheimer’s disease showed lower HDL-C/TC (%) around 25.3% than the control, at around 31.2% [21]. On the other hand, it has been proposed recently that HDL particle number (HDL-P) may be a better biomarker of CVD risk than HDL-C [22]. Taken together, the percentage of HDL-C, and the size of HDL particles and HDL-P, are emerging biomarkers as predictors of CVD via the impairment of HDL functionality, including anti-oxidant and anti-inflammatory activity [23].

On the other hand, the severity of Alzheimer’s disease is highly correlated with decreased HDL-C and serum apoA-I, as reported by Siest group [24] in the French population. HDL-C (mmol/L) was significantly lower in the AD group, and calculated HDL-C/TC (%) was also significantly decreased in the AD group (18.9%) compared with control group (24.5%), although the AD group showed 7.8% lower TC than the control group. In the same context, the InChianti study with an Italian population [25] also showed that HDL-C/TC (%) was significantly lowered in the dementia group (HDL-C/TC (%), 24.8%) compared with the control group (HDL-C/TC (%), 25.6%).

It has been established that keeping BP in the normal range is an effective strategy for the prevention of primary and secondary dementia. HDL has numerous beneficial functions including preventing amyloid beta aggregation [26] and protecting against amyloid beta-induced inflammation [27]. However, there has been no study to elucidate the correlation between blood pressure and the HDL-C level in the adult population aged 20–80 years. The Tromsø study showed that biological interrelations between BP and serum lipids [28], TC and non-HDL-C levels increased significantly with the elevation of SBP and DBP in both genders. However, the study excluded the elderly population; including only men 20–54 years old and women 20–49 years old. Regarding low HDL-C and high-normal blood pressure (HNBP, SBP 130–139 or DBP 85–89 mmHg), individuals in the elderly Korean population with HNBP showed a significantly increased risk of all-cause mortality, especially when combined with low HDL-C [29].

Regarding mechanistic insight, it has been reported that an excess of very low-density lipoproteins (VLDL) can bind to scavenger receptor B-I (SRB-I), which is a cholesterol docking receptor in the mitochondria to result in more production of aldosterone via acute or sustained stimulation of signaling [30,31]. The link between obesity, hypertension and aldosterone could explain the mechanism for lowered HDL-C and elevated BP with increasing age. Therefore, it can be supposed that a lowered HDL-C might allow more binding of VLDL or LDL to the SR–BI receptor, resulting in an elevation in BP. High LDL-C/HDL-C ratio was also associated with cardiovascular events in patients with acute coronary syndrome [32,33]. Moreover, it has been reported that the TG/HDL-C ratio was related to arterial stiffness and blood pressure [34]. The complete Seventh Report of the Joint National Committee on Prevention, Detection, Evaluation, and Treatment of High Blood Pressure [35] coincides with our current finding that SBP usually increases in both men and women aged between 20 and 80 years old. However, in the current study, DBP gradually increased from 70 mmHg between 20–30 years, until midlife, when it reached 80 mmHg in the subjects’ 50s, then decreased back to 70 mmHg in their 80s.

## 5. Conclusions

SBP increased with age from 20 to 80 years in total population, but DBP did not. The elevation of SBP is well correlated with a decrease in HDL-C during the entire life. The ratio of HDL-C in TC (HDL-C/TC (%)) was more closely and significantly associated with changes in SBP and DBP in both genders. Women showed a sharper decrease in HDL-C, correlating with increasing SBP, after menopause.

## Figures and Tables

**Table 1:** General characteristics of total population in this study from Korean national health and nutrition examination survey 2017.

Characteristics	Men *N* = 2017 (Mean ± SD)	Women *N* = 2535 (Mean ± SD)	* *p*	Total *N* = 4552 (Mean ± SD)
SBP (mmHg)	119.2 ± 14.7	113.6 ± 16.1	<0.001	116.1 ± 15.8
DBP (mmHg)	78.2 ± 10.2	73.4 ± 9.4	<0.001	75.5 ± 10.0
HDL-C (mg/dL)	47.6 ± 11.0	55.6 ± 12.2	<0.001	52.0 ± 12.3
TC (mg/dL)	195.6 ± 36.6	197.4 ± 36.9	0.103	196.6 ± 36.8
TG (mg/ dL)	158.5 ± 129.1	107.3 ± 73.1	<0.001	130.0 ± 104.9
HDL-C/TC (%)	25.0 ± 6.9	28.9 ± 7.1	<0.001	27.2 ± 7.3
LDL-C (mg/dL)	116.3 ± 34.9	120.4 ± 32.5	0.00006	118.6 ± 33.6
LDL-C/HDL-C	2.5 ± 0.9	2.3 ± 0.8	<0.001	2.4 ± 0.9
TG/HDL-C	3.8 ± 4.3	2.2 ± 2.1	<0.001	2.9 ± 3.4
Age (year)	47.6 ± 15.7	47.6 ± 15.0	0.988	47.6 ± 15.4

Data are expressed as mean ± SD (standard deviation) or N (%). SBP, systolic blood pressure; DBP, diastolic blood pressure; HDL-C, high-density lipoprotein-cholesterol; TC, total cholesterol; TG, triglyceride; HDL-C/TC (%), HDL-C/TC *100; LDL-C, low-density lipoprotein-cholesterol. * *p* value for difference between men and women (*p* < 0.05).

**Table 2:** Distribution of SBP, DBP, age and HDL-C in men and women from Korean national health and nutrition examination survey 2017.

Group	Covariates	SBP (mmHg)				DBP (mmHg)					
		≤119	120–139	140–159	≥160	≤79	80–89	90–99	≥100	* *p*	^￥^ *p*
Men *N* = 2017 (Mean ± SD)	*N* (%)	1134 (56.2)	699 (34.7)	159 (7.9)	25 (1.2)	1158 (57.4)	627 (31.1)	177 (8.8)	55 (2.7)		
	Age (year)	45.2 ± 15.1	49.0 ± 15.9	56.8 ± 15.2	60.5 ± 13.9	48.5 ± 17.2	46.6 ± 13.9	46.8 ±13.0	44.0 ± 8.3	<0.001	0.023
	HDL-C (mg/dL)	47.5 ± 10.6	47.5 ± 11.1	48.5 ± 13.0	47.2 ± 12.3	47.2 ± 10.4	48.3 ± 11.5	47.3 ± 11.8	48.8 ± 13.7	0.764	0.196
	HDL-C/TC (%)	25.4 ± 6.7	24.5 ± 7.0	24.5 ± 6.9	25.3 ± 7.5	25.6 ± 6.9	24.4 ± 6.7	23.7 ± 6.8	24.0 ± 7.4	0.025	0.0001
Women *N* = 2535 (Mean ± SD)	*N* (%)	1771 (69.9)	581 (22.9)	154 (6.1)	29 (1.1)	1940 (76.5)	462 (18.2)	112 (4.4)	21 (0.8)		
	Age (year)	43.2 ± 13.6	56.9 ± 13.3	60.5 ± 12.1	62.7 ± 8.9	46.3 ± 15.6	51.6 ± 12.7	51.9 ± 10.8	52.5 ± 14.0	<0.001	<0.001
	HDL-C (mg/dL)	56.4 ± 12.0	54.2 ± 12.3	51.8 ± 12.3	53.3 ± 11.9	55.7 ± 12.0	55.3 ± 12.6	55.1 ± 13.4	54.0 ± 11.5	<0.001	0.820
	HDL-C/TC (%)	29.8 ± 6.9	27.1 ± 7.2	25.7 ± 7.1	25.8 ± 7.9	29.4 ± 6.9	27.3 ± 7.5	27.2 ± 7.7	27.1 ± 8.6	<0.001	<0.001
	^†^ *p*	0.0003	<0.001	0.017	0.485	0.0006	<0.001	0.0003	0.015		
	^₸^ *p*	<0.001	<0.001	0.019	0.069	<0.001	<0.001	<0.001	0.127		
	^€^ *p*	<0.001	<0.001	0.139	0.837	<0.001	<0.001	<0.001	0.120		
Total *N* = 4552 (Mean ± SD)	*N* (%)	2905 (63.8)	1280 (28.1)	313 (6.9)	54 (1.2)	3098 (68.1)	1089 (23.9)	289 (6.3)	76 (1.7)		
	Age (year)	43.9 ± 14.2	52.6 ± 15.2	58.6 ± 13.9	61.7 ± 11.4	47.1 ± 16.2	48.7 ± 13.6	48.8 ± 12.4	46.3 ± 10.8	<0.001	0.012
	HDL-C (mg/dL)	52.9 ± 12.3	50.5 ± 12.1	50.1 ± 12.7	50.5 ± 12.4	52.5 ± 12.1	51.2 ± 12.5	50.3 ± 13.0	50.2 ± 13.3	<0.001	0.0009
	HDL-C/TC (%)	28.1 ± 7.2	25.7 ± 7.2	25.1 ± 7.0	25.5 ± 7.7	28.0 ± 7.1	25.6 ± 7.2	25.1 ± 7.3	24.8 ± 7.8	<0.001	<0.001

Data are expressed as mean ± SD (standard deviation) or *N* (%). SBP, systolic blood pressure; DBP, diastolic blood pressure; HDL-C, high-density lipoprotein-cholesterol; HDL-C/TC (%), HDL-C/TC *100. ^†^*p* value for difference between men and women depending on age (*p* < 0.05). ^₸^ *p* value for difference between men and women depending on HDL-C (*p* < 0.05). ^€^ *p* value for difference between men and women depend on HDL-C/TC (%) (*p* < 0.05). * *p*-value across age, HDL-C, HDL-C/TC (%) of SBP depends on gender (*p* < 0.05). ^￥^ *p*-value across age, HDL-C, HDL-C/TC (%) of DBP depends on gender (*p* < 0.05).

**Table 3:** Distribution of SBP, DBP, HDL-C, HDL-C/TC (%) depends on age from Korean national health and nutrition examination survey 2017.

Group	Age	20–29	30–39	40–49	50–59	60–69	70–79	80	* *p*
Men *N* = 2017 (Mean ± SD)	*N* (%)	303 (15.0)	388 (19.2)	436 (21.6)	392 (19.4)	287 (14.2)	168 (8.3)	43 (2.1)	
	SBP (mmHg)	114.9 ± 11.1	116.6 ± 12.0	117.3 ± 13.8	120.9 ± 15.3	122.8 ± 16.3	125.4 ± 17.1	129.3 ± 20.4	<0.001
	DBP (mmHg)	75.0 ± 9.3	79.7 ± 9.9	80.7 ± 10.4	80.7 ± 9.5	76.4 ± 9.5	73.0 ± 9.2	70.6 ± 10.5	<0.001
	HDL-C (mg/dL)	50.0 ± 11.2	47.3 ± 10.3	47.4 ± 10.5	47.9 ± 11.4	47.1 ± 11.5	45.6 ± 10.5	43.7 ± 11.5	0.0002
	HDL-C/TC (%)	27.6 ± 7.2	24.3 ± 6.5	23.6 ± 6.3	24.7 ± 6.7	25.5 ± 7.2	25.8 ± 7.1	25.0 ± 6.7	<0.001
Women *N* = 2535 (Mean ± SD)	*N* (%)	340 (13.4)	481 (19.0)	594 (23.4)	536 (21.1)	367 (14.5)	172 (6.8)	45 (1.8)	
	SBP (mmHg)	104.5 ± 9.0	106.3 ± 11.0	110.4 ± 13.6	117.4 ± 16.9	123.2 ± 17.0	127.9 ± 17.4	127.3 ± 14.6	<0.001
	DBP (mmHg)	69.0 ± 7.7	71.4 ± 8.7	74.1 ± 9.0	76.7 ± 9.5	74.8 ± 9.5	72.8 ± 10.4	69.0 ± 9.8	<0.001
	HDL-C (mg/dL)	58.4 ± 11.3	57.7 ± 12.4	56.0 ± 12.4	55.5 ± 12.0	52.4 ± 11.4	51.1 ± 11.7	49.3 ± 10.7	<0.001
	HDL-C/TC (%)	32.8 ± 6.3	31.0 ± 7.3	28.8 ± 6.8	27.0 ± 6.5	26.5 ± 6.6	26.8 ± 7.5	26.5 ± 6.8	<0.001
	^†^ *p*	<0.001	<0.001	<0.001	0.0014	0.732	0.183	0.603	
	^₸^ *p*	<0.001	<0.001	<0.001	<0.001	0.04	0.819	0.473	
	^€^ *p*	<0.001	<0.001	<0.001	<0.001	<0.001	0.000006	0.022	
	^ǂ^ *p*	<0.001	<0.001	<0.001	<0.001	0.058	0.208	0.316	
Total *N* = 4552 (Mean ± SD)	*N* (%)	643 (14.1)	869 (19.1)	1030 (22.6)	928 (20.4)	654 (14.4)	340 (7.5)	88 (1.9)	
	SBP (mmHg)	109.4 ± 11.3	110.9 ± 12.6	113.3 ± 14.1	118.9 ± 16.3	123.0 ± 16.7	126.6 ± 17.3	128.3 ± 17.6	<0.001
	DBP (mmHg)	71.8 ± 9.0	75.1 ± 10.1	76.9 ± 10.2	78.4 ± 9.7	75.5 ± 9.5	72.9 ± 9.8	69.8 ± 10.1	<0.001
	HDL-C (mg/dL)	54.4 ± 12.0	53.1 ± 12.6	52.4 ± 12.4	52.3 ± 12.3	50.1 ± 11.7	48.4 ± 11.5	46.6 ± 11.3	<0.001
	HDL-C/TC (%)	30.4 ± 7.2	28.0 ± 7.7	26.6 ± 7.1	26.0 ± 6.7	26.1 ± 6.9	26.3 ± 7.3	25.8 ± 6.8	<0.001

Data are expressed as mean ± SD (standard deviation) or N (%). SBP, systolic blood pressure; DBP, diastolic blood pressure; HDL-C, high-density lipoprotein-cholesterol; %HDL-C, HDL-C/TC*100. ^†^ *p* value for difference between men and women depend on SBP (*p* < 0.05). ^₸^ *p* value for difference between men and women depend on DBP (*p* < 0.05). ^€^ *p* value for difference between men and women depend on HDL-C (*p* < 0.05). ^ǂ^ *p* value for difference between men and women depend on %HDL-C (*p* < 0.05). * *p*-value across SBP, DBP, HDL-C, %HDL-C of age depends on gender (*p* < 0.05).

**Table 4:** Regression analysis between HDL-C and SBP or DBP in total population.

Dependent Variable	Independent Variable	Standard Error	β	t-Value	*p*-Value	VIF
SBP	HDL-C	0.019	−0.148	−7.880	<0.001 ***	1.000
	R = 0.116, R^2^ = 0.013, Adjusted R^2^ = 0.013 F = 62.088, *p* < 0.001, Dublin-Watson = 1.132					
DBP	HDL-C	0.012	−0.065	−5.376	<0.001 ***	1.000
	R = 0.079, R^2^ = 0.006, Adjusted R^2^ = 0.006 F = 28.900, *p* < 0.001, Dublin-Watson = 0.560					

SBP, systolic blood pressure; DBP, diastolic blood pressure; HDL-C, high-density lipoprotein-cholesterol. VIF, variance inflation factor. *** *p* < 0.001.
